# Increasing access to microfluidics for studying fungi and other branched biological structures

**DOI:** 10.1186/s40694-019-0071-z

**Published:** 2019-06-10

**Authors:** Larry J. Millet, Jayde Aufrecht, Jessy Labbé, Jessie Uehling, Rytas Vilgalys, Myka L. Estes, Cora Miquel Guennoc, Aurélie Deveau, Stefan Olsson, Gregory Bonito, Mitchel J. Doktycz, Scott T. Retterer

**Affiliations:** 1 Biosciences Division, Oak Ridge National Laboratory, PO Box 2008, MS 6445, Oak Ridge, TN 37831 USA; 2 The Bredesen Center, University of Tennessee-Knoxville, Knoxville, TN 37996 USA; 3 The Center for Nanophase Materials Sciences, Oak Ridge National Laboratory, PO Box 2008, MS 6445, Oak Ridge, TN 37831 USA; 4 Department of Biochemistry and Cellular and Molecular Biology, The University of Tennessee, Knoxville, TN 37996 USA; 5 Biology Department, Duke University, Box 90338, Durham, NC 27708 USA; 6 Department of Plant and Microbial Biology, University of California at Berkeley, Berkeley, CA 94703 USA; 7 The Center for Neuroscience, University of California Davis, One Shields Avenue, Davis, CA 95618 USA; 8 Institut national de la recherche agronomique (INRA), Centre INRA-Lorraine, 54280 Champenoux, France; 9 Fujian Agricultural and Forestry University, Fuzhou City, 350002 Fujian Province China; 10 Department of Plant, Soil and Microbial Sciences, Michigan State University, East Lansing, MI 48824 USA

**Keywords:** Fungi, Microfluidics, Cell culture, Bacterial-fungal interactions, Plant root, *Arabidopsis*, Neuron, Branching biology

## Abstract

**Background:**

Microfluidic systems are well-suited for studying mixed biological communities for improving industrial processes of fermentation, biofuel production, and pharmaceutical production. The results of which have the potential to resolve the underlying mechanisms of growth and transport in these complex branched living systems. Microfluidics provide controlled environments and improved optical access for real-time and high-resolution imaging studies that allow high-content and quantitative analyses. Studying growing branched structures and the dynamics of cellular interactions with both biotic and abiotic cues provides context for molecule production and genetic manipulations. To make progress in this arena, technical and logistical barriers must be overcome to more effectively deploy microfluidics in biological disciplines. A principle technical barrier is the process of assembling, sterilizing, and hydrating the microfluidic system; the lack of the necessary equipment for the preparatory process is a contributing factor to this barrier. To improve access to microfluidic systems, we present the development, characterization, and implementation of a microfluidics assembly and packaging process that builds on self-priming point-of-care principles to achieve “ready-to-use microfluidics.”

**Results:**

We present results from domestic and international collaborations using novel microfluidic architectures prepared with a unique packaging protocol. We implement this approach by focusing primarily on filamentous fungi; we also demonstrate the utility of this approach for collaborations on plants and neurons. In this work we (1) determine the shelf-life of ready-to-use microfluidics, (2) demonstrate biofilm-like colonization on fungi, (3) describe bacterial motility on fungal hyphae (fungal highway), (4) report material-dependent bacterial-fungal colonization, (5) demonstrate germination of vacuum-sealed *Arabidopsis* seeds in microfluidics stored for up to 2 weeks, and (6) observe bidirectional cytoplasmic streaming in fungi.

**Conclusions:**

This pre-packaging approach provides a simple, one step process to initiate microfluidics in any setting for fungal studies, bacteria-fungal interactions, and other biological inquiries. This process improves access to microfluidics for controlling biological microenvironments, and further enabling visual and quantitative analysis of fungal cultures.

**Electronic supplementary material:**

The online version of this article (10.1186/s40694-019-0071-z) contains supplementary material, which is available to authorized users.

## Background

Morphological branching is a common and fundamental mode of biological propagation and growth [1–4]. Branched biological structures are evident across all taxonomic kingdoms and size scales. Aggregates of unicellular bacteria form branching communities, which gives rise to colonial phenotypes that are often distinct (e.g. morphology and color) [5–10]. From roots to branches, and stems to leaf structures; plants arborize to develop high surface area tissues; a plants branching structure is vital for nutrient absorption in photosynthesis, energy transport and storage, reproduction, and waste release [11]. Hyphal branching in fungi is involved in beneficial and detrimental interactions among plants and microbes, it is through these high surface-area structures that nutrient uptake, environmental signaling, and communication are achieved [12–14]. Neuronal cells and tissues branch to connect with targets cells and organs for efficient computation of information and the coordination of physiological processes [15, 16]. Understanding the physical and molecular cues that initiate the formation and function of branching structures and resolve the underlying mechanisms of growth and transport in branched tissues will benefit relevant industries including those involved in fermentation, biofuel production, and health care. To capture the dynamics of this process at such a fine spatial scale requires a culturing platform that enables real-time and high-resolution imaging. While microtechnological methods are well established for culturing neurons and mammalian cells, advancements for increasing the precision and sophistication for measuring plant, fungal, and microbial structures and dynamics (e.g. growth, forces, secretions) are in demand [17–21].

Microfluidic platforms are particularly well-suited for guiding, restraining, and imaging branched structural growth and specification; through a modular on-chip approach, branched biological systems can be interrogated in ways not possible via conventional culture. For example, customized microfluidic designs with interconnected compartments may be used to facilitate and isolate desired branching features. Highly-controlled microfluidic environments also improve the ability to study innate mechanisms and environmental influences guiding process outgrowth, cellular physiology, and biological interactions [22–26]. Despite these benefits, the intersection of microfluidics and biology is highly interdisciplinary and often requires strong cross-departmental or multi-institutional collaborations in order to apply the precision technology toward answering deeply-rooted biological questions. Microfluidic applications for rhizosphere-on-a-chip are providing high-resolution access for studying the dynamics of root-bacterial interactions [27–29]. Recent studies on fungal polarity and growth, and plant development and metabolism demonstrate the ability to increase throughput and semi-automate work to resolve molecular regulations of mesoscale biological interactions using microfluidics [30, 31].

Despite the numerous papers that have sought to simplify microfluidics use for biological inquiry [32, 33], technical and logistical barriers still exist that counteract the effective deployment of microfluidics in biological disciplines. Technical barriers to bio-microfluidic collaborations include equipment, such as vacuum pumps, house vacuum systems, syringe pumps, fabrication facilities. Physical barriers include institutional proximity. Unfortunately, the lack of familiarity with microfluidic systems (creation and implementation) and personal contacts are also primary impediments to initiating collaborations. Overcoming these limitations will promote the broad use of a microfluidics tool-set for biological inquiry. For example, microfluidic platforms can readily be designed to provide an environment that allows for resolving the biological response to local physical, chemical, and biological cues integrated within the platform design [34–37]. Complex barriers, topographical cues, compartmentalization of chemically and physically interacting biological systems, and complex chemical gradients can be controlled and manipulated within microfluidic systems to replicate natural interactions and conditions in a fundamental form [22, 38–42].

Assembling, sterilizing, and hydrating the microsystem is one of the principle technical barriers that limits the use and implementation of microfluidics in biological investigation. The lack of both simple and precision equipment is a key contributing factor that further complicates the use of microfluidics [43]. In the effort to help collaborators overcome barriers to implementing microfluidics, we have resolved a preparation strategy that greatly minimizes the hurdles associated with using microfluidics in biology. Here, we present the development, characterization, and implementation of a microfluidics assembly process that builds on principles employed in self-priming and point-of-care technologies [44–49]. We designed and implemented novel microfluidic chips, and also tested previously implemented plant-chip platforms [29], toward validating the utility of ready-to-use packaging to overcome common implementation barriers for environmental science research and for new users of microfluidics. We believe that this approach is applicable for a variety of different platform architectures and a wide range of biological studies. The result of our fabrication process is a sterile, ready-to-use microfluidic system that can be implemented anywhere. We demonstrate initial results from international collaborations focusing on the biology of filamentous fungi and include other applications for branching samples such as plants and neurons. In these biological systems, microfluidics enable real-time visualization and quantification of fungi, plants and multispecies interactions.

## Methods

**Fig. 1:**
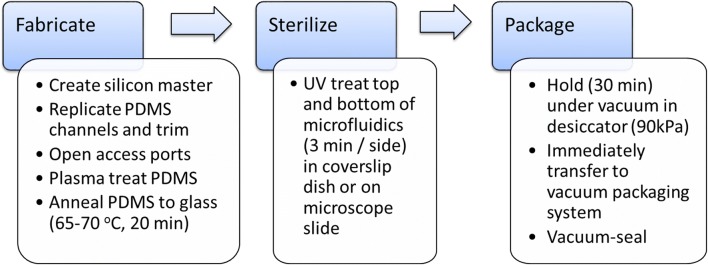
An overview of the fabrication and assembly protocol for creating ready-to-use microfluidics. Standard photolithography is used to create microfluidic masters, conventional replicate molding with PDMS is used to produce microfluidic structures that are annealed to glass slides or coverslips. UV light exposure or autoclaving processes are used to sterilize the microfluidic platforms. Equilibration under vacuum is the conditioning step that immediately precedes vacuum packaging to produce a ready-to-use microfluidic platform

Methods for the fabrication and assembly of microfluidics are detailed in Additional file 1. Figure 1 summarizes the fabrication and assembly process used to prepare ‘ready-to-use microfluidics.’

### Vacuum packaging

**Fig. 2:**
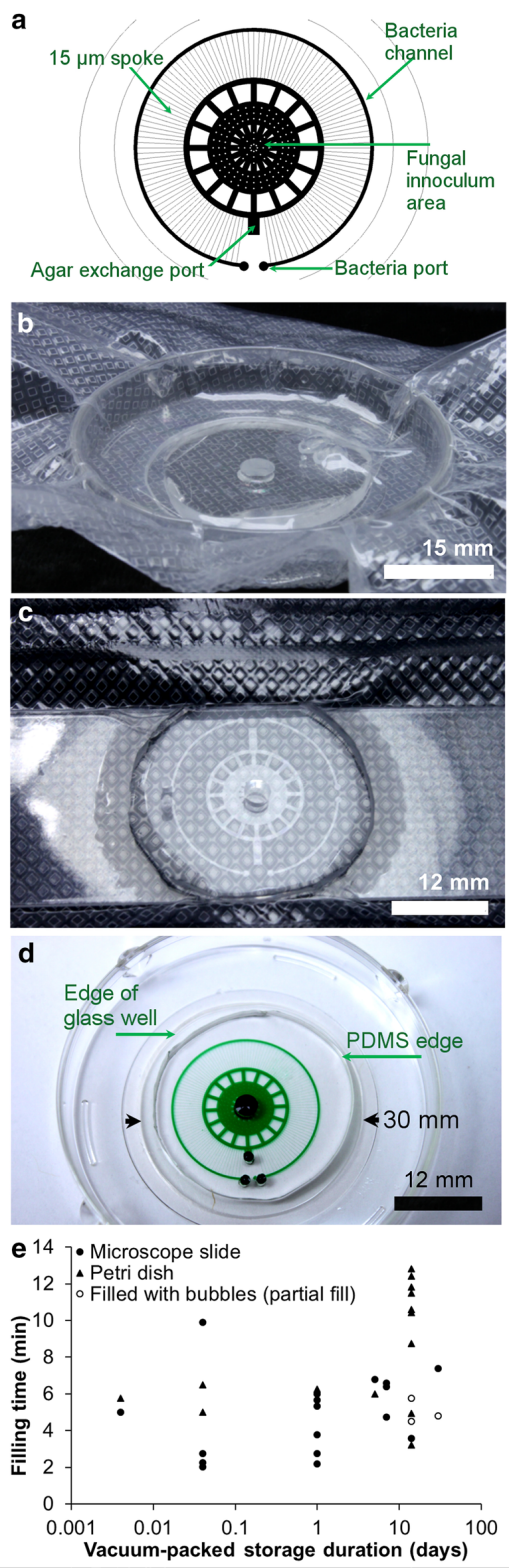
Sterile pre-packaged ready-to-use microfluidics. **a** Design of the spoke-wheel device used for characterizing the vacuum-packaging and use process, and for quantifying the ‘best if used by date.’ **b** Spoke wheel microfluidic chamber with integrated PDMS tubing in a glass-bottom dish, vacuum-equilibrated (20 min), then vacuum-sealed for storage, transport, or direct use. **c** Autoclaved microscope slide with spoke-wheel microfluidic culture chamber in a vacuum-sealed pouch. **d** The spoke wheel microfluidic device in a coverslip-bottom dish filled with water containing green food-dye. **e** Vacuum packaged microfluidics on microscope slides or in coverslip-bottom dishes maintain ability to fill (< 2 weeks) within 13 min

Fully assembled microfluidic channels on glass slides, or in glass-bottom dishes, were first equilibrated under vacuum (90 kPa, 30 min) in a sealed glass desiccator. Then, channel assemblies were immediately removed from the vacuum chamber and transferred to thermal-sealable pouches and immediately vacuum-sealed (82 kPa, Food Saver model #V3240) for transport and until the time of use. To determine the ‘best if used by date’, 35 microfluidic channel samples were vacuum packed and marked with the test date. To measure the time for the microfluidics to fill, channels were removed from vacuum-sealed pouches and loaded with water containing food color dye; the time from dye addition to channel fill was documented and plotted (Fig. 2).

### Microfluidic design and operation

*Spoke*-*wheel microfluidics* This novel chip architecture (new and not previously published) consists of a central circular chamber (8 mm) with the roof supported by pillars (*n* = 118 *dia.* = 100 µm). Sixteen primary radiating channels (*l* = 1.5 mm, *w* = 500 µm) emanate from the central chamber to terminate at a primary concentric channel (*r* = 6 mm, *w* = 500 µm). Secondary radial channels (*n* = 123, *dia*. = 4 mm) connect the primary and secondary concentric channels. The depth of the entire channel system is different for fungal cultures (*h* = 11 µm) and neuronal cultures (*h* = 50 µm).

Two ports at the ends of the peripheral channel permit fluidic exchange. The middle ‘accessory’ port between the center well and the peripheral ports allows for efficient priming of the microfluidic space; for bacterial-fungal interaction studies, this port is for loading agar into the central chamber. For bacterial-fungal interactions, the center well is used for inoculating fungi, the primary concentric channel is used for observing bacterial-fungal interactions, and the peripheral channel is used for inoculating bacteria. For neuronal cultures, the center well is used for the introduction of dissociated neurons, the peripheral channel ports are used for media exchange.

*“ORNL” chambers* This novel architecture (new and not previously published) provides two parallel boundary channels that connect two separate ports. Parallel boundary channels are connected through a grid of microfluidic channels (*w* = 15 µm, *h* = 17 µm). Within the grid between the boundary channels are four chambers formed of the letters “ORNL.” Pitch of the 15-µm channel grid is ~ 500 µm for the smaller array. This design enables fungal growth in chambers for hyphal isolation and bacterial fungal interaction studies.

*Root chip microfluidic* This microfluidic architecture was previously designed for *Arabidopsis* seed germination and growth rate measures, and has been published for root-bacteria interaction studies [29, 50]. Here, this device is used to demonstrate that prior art, such as the seed-in-chip system, can be incorporated into this fabrication process for decreasing barriers to implementing microfluidics in collaborations. Channel dimensions include a central channel (4 mm *l*, 200 µm *w*, 200 µm *h*) guides the primary root shaft down a larger (20 µm tall, 1 µL) rectangular chamber for bathing the growing root hairs. The PDMS microfluidic chip was exposed to air plasma prior to conformal contact and thermal annealing (70 °C). The channel-on-slide structures were cleaned by autoclave sterilization prior to vacuum-packaging of Arabidopsis seeds.

### Cell cultures

Protocols for preparations and cell cultures of eukaryotic and prokaryotic cells (fungi, plant, bacteria, and neuronal cultures) are modified from prior reports and detailed in supplementary information [51–64].

## Results

The fabrication and assembly of PDMS-based microfluidics for fungi, roots, and neurons was achieved through conventional photolithographic processes yielding PDMS-channels on microscope slides or coverslip-bottomed dishes. UV-treatment (for glass-bottomed dishes), or autoclave sterilization (microscope slides), produces a sterile microfluidic platform that is amenable for cell culture studies (Fig. 1). Figure 2a–d shows that microfluidics on glass-bottomed dishes (with or without lids) or microscope slides can be vacuum sealed in plastic pouches for shipping, and later, fluidic priming. Low viscosity solutions (e.g. culture media or water) completely fill the entire channel network within minutes after removing the PDMS spoke-wheel microfluidics from the vacuum chamber. Figure 2e shows that vacuum-packaged microfluidics continue to fill with culture media toward completion, if used within 14 days of the vacuum sealing date.

**Fig. 3:**
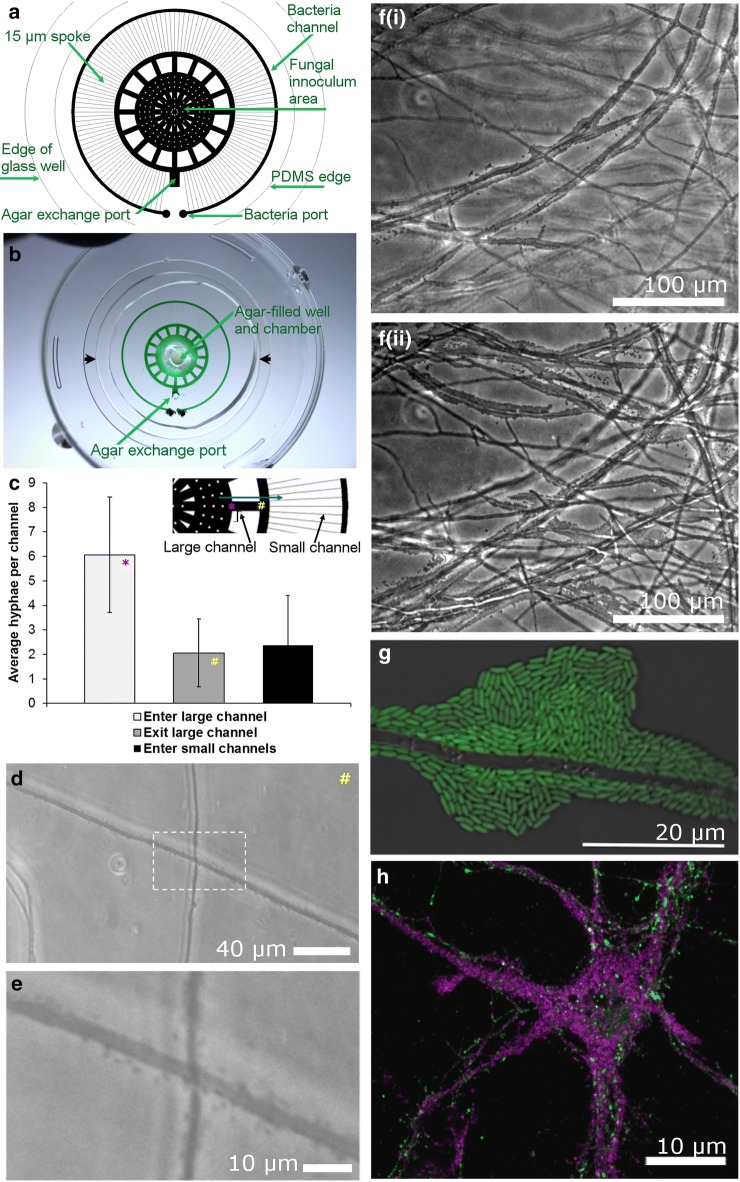
Microfluidics for bacterial-fungal interaction studies. **a** The spoke-wheel microfluidic design incorporates ports for separate, spatially-defined inoculations of fungi and bacteria. **b** Shown are green dye-filled channels (loaded with vacuum-assisted filling) in a glass-bottomed dish. An agar plug fills the center of the chamber introduced through the center culture well and aspirated through the agar exchange port. The agar plug holds the fungal inoculum in place and minimizes dehydration during growth. For scale, the open glass surface (between black arrows) is 30 mm. **c** Average number of hyphae per primary and secondary radiating channels from a single dish (15 DIV). The inset image shows channel locations that correspond to the graphed data. **d**, **e** Bacterial-fungal interactions are conveniently established and imaged with microfluidic systems. Image **d** and inset **e** of *Pseudomonas fluorescens* GM41 navigating the *Laccaria bicolor* fungal highway within a microfluidic chamber (3 DIV bacteria co-culture). **f**(i, ii) *P. fluorescens* GM41 bacterial communities accumulate where fungi contact the glass (i) or PDMS (ii) surface, preferentially forming at the PDMS-hyphae surface **d** over the glass-hyphae interface (30 DIV co-culture). **g** In the same device architecture, *Pseudomonas fluorescens* BBc6 biofilm-like accumulation on ectomycorrhizal fungi (*L. bicolor* S238N) 16 h after bacterial inoculation. **h** Vacuum-packed spoke-wheel microfluidics are permissive for even the most sensitive cell cultures, neurons. Here, neurons (DIV 4) were transfected for molecular imaging studies (VAMP2, magenta; PSD-95, green)

Prior to distributing vacuum-packed microfluidics to collaborators, we characterized the feasibility of our spoke-wheel microfluidic design to permit the observation of bacterial-fungal interactions for high resolution imaging studies. Figure 3a, b shows a spoke-wheel microfluidic architecture that promotes fungal-bacterial interaction studies in microfluidics. The agar-filled central chamber promotes fungal growth while stabilizing the fungal inoculum during culture and shipping (Fig. 3b). The peripheral concentric channel enables the introduction of bacteria at the periphery of the microenvironment. Center to edge, the radiating channels maintain a similar culture space and volume in the channels while providing increasingly narrow passages for the navigation of the fungal hyphae. The central chamber (without the culture well) has a volume of 0.04 µL, the 16 primary radiating channels (large channels) with the primary concentric channel retains 0.03 µL, and the sum of the 63 spokes (small channels) is 0.04 µL. Figure 3c shows a summary of the number of hyphae that colonize the intersection of the spoke-wheel of the platform following 2 weeks in culture. In Fig. 3c, an asterisk and hash symbol in the inset and data bar demarcate the corresponding values for regions of the device quantified as entering and exciting the large microfluidic channel prior to entering the small microfluidic spoke channel. Figure 3d, e is an example of hyphae in the large microfluidic channel, on average two hyphae occupy the intersection of the primary concentric circle and the hyphal spoke to permit a low-density of hyphae for resolving bacteria-fungal interactions. As the fungal cultures continue to grow and mature, the fungal hyphae reproducibly fill the device.

After 3 days of co-culturing bacteria in the microfluidic environment, individual hyphae and bacterial colonizations can be observed in the 15 µm-wide radiating (spoke) channel (Additional file 2: Figure S1). After 4 weeks in culture, fungal hyphae navigate the entire fluidic architecture with bacteria to intersect in open places and align in confined spaces. Probiotic biofilms of the mutualistic *Pseudomonas fluorescens* GM41 become established on the surface of *Laccaria bicolor* hyphae [65, 66]. We observed that *L. bicolor* hyphae show preference for the PDMS surface over the glass surface (Fig. 3f(i–ii)), which is softer and more gas permeable. The bacterial colonies form at either glass or PDMS interfaces, but colony prevalence is substantially reduced at the glass-fluid interface (Fig. 3f(i)) as compared to the PDMS-fluid interface (Fig. 3f(ii)). Through collaboration, high-resolution images of fluorescent *P. fluorescens* BBc6 colonization of fungal hyphae were observed (Fig. 3g).

In minimal media, *P. fluorescens* GM41 cells navigate along the surface of *Laccaria bicolor* S238N hyphae in vitro prior to colonization on or adjacent to the hyphae (Additional file 3: Figure S2). A standard deviation image of a an image stack (25 frames) from a time-lapse image series (6 s) shows a range of densities of bacteria (white borders on fungi against a black background) moving along *Laccaria* hyphae (Additional file 3: Figure S2). Kymographs (line traces over time displayed in a two-dimension image) clarify the range of densities for which bacterial transport occurs on adjacent segments of the hypha (Additional file 3: Figure S2). But the biological question remains to be answered exactly how bacteria select colonization sites on fungal hyphae. While fungal highway and bacterial-fungal interactions can be imaged outside of microfluidics, these microsystems make the process more convenient by preventing drying and enabling confinement over long culture periods to image and map bacterial motility and colonization on hyphal outgrowths.

With this vacuum packaging process being amenable for coverslip-bottomed dishes, with or without lids, we prepared 22 dishes capped with lids in vacuum-sealed pouches. Nineteen dishes were shipped to collaborators for culturing cortical neurons for transfection and molecular imaging of neuronal connections; three dishes were retained in-house. Of the 22 coverslip-bottomed dishes, three coverslips buckled due to the vacuum pressure and pouch pressing on the back of the fragile coverslip window. Placing the glass-bottomed dish within the lid of a 50 mm Petri dish stabilized the glass and solved the issue of the collapsing cover glass. With vacuum-packaged microfluidics, collaborators that are new to microfluidics were able to achieve neuronal cultures in challenging culture volumes and microfluidic dimensions (Fig. 3h).

**Fig. 4:**
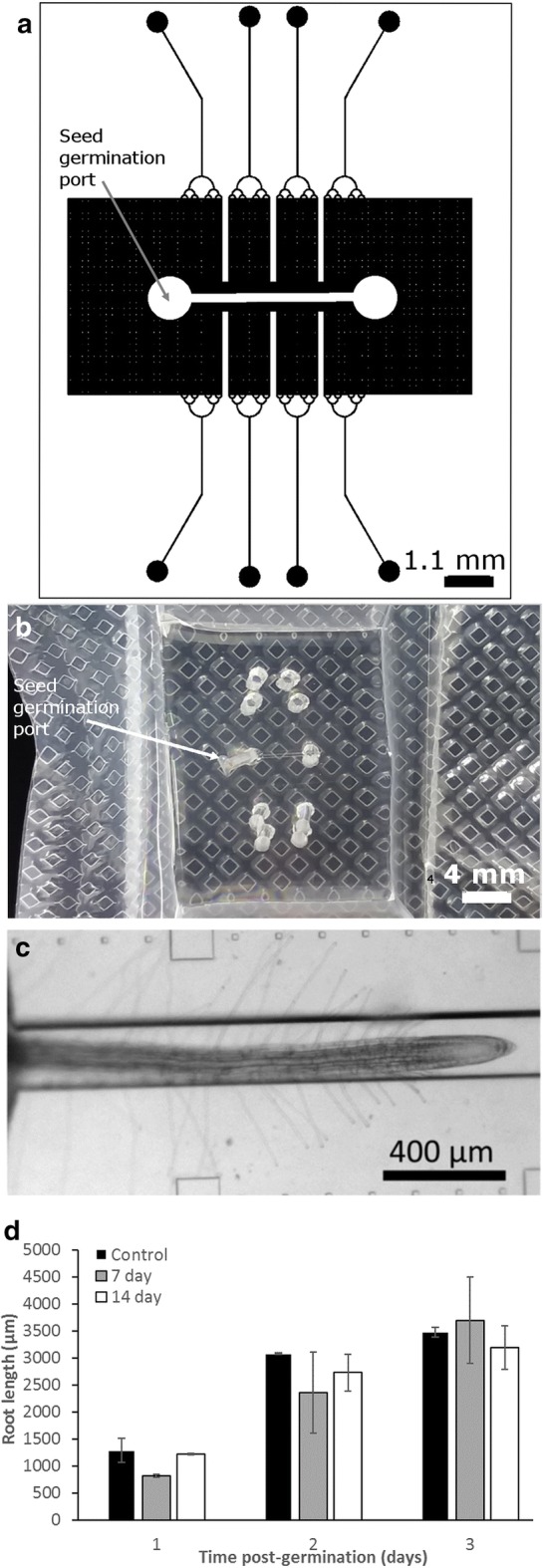
Root-chip microfluidic channel for packaging, storing, culturing, and imaging *Arabidopsis* roots. **a** The chip design contains a single root culture channel and accessory ports for sampling and delivery of media and microbes. **b** A vacuum-packaged microfluidic root-chip shown with the same orientation. **c** *Arabidopsis* root growing in the central microfluidic channel with marks for measuring growth. **d** Growth data for vacuum-packed and control seeds. The packaging process does not attenuate growth conditions of stored roots (< 14 days at room temperature)

Figure 4a–d shows seed germination results for *Arabidopsis thaliana* seeds cultivated in a root-chip system. In prior work, this system supported bacterial-plant interaction studies; here, we vacuum packed *A. thaliana* seeds in the system to determine if the packaging process negatively influenced the seed germination process. After 7 (n = 3) and 14 (n = 7) days of storage in the vacuum packaging (kept in the dark), 100% of the seeds germinated within 3 days of opening the package and filling the chamber with Murashige-Skoog medium (Fig. 4d). Of these germinated seedlings, all of the roots from the 7-day storage and two of the roots from the 14-day storage test grew into the large microfluidic channel. By the second and third days in vitro, the growth rate of the roots for the vacuum-stored seedlings in the channel were not significantly different from the growth rate of non-vacuumed control (n = 3) seedlings (Fig. 4d), although initial growth rates at day 1 were significantly different (*p* = 0.0227), unpaired *t* test, *p* < 0.05.

**Fig. 5:**
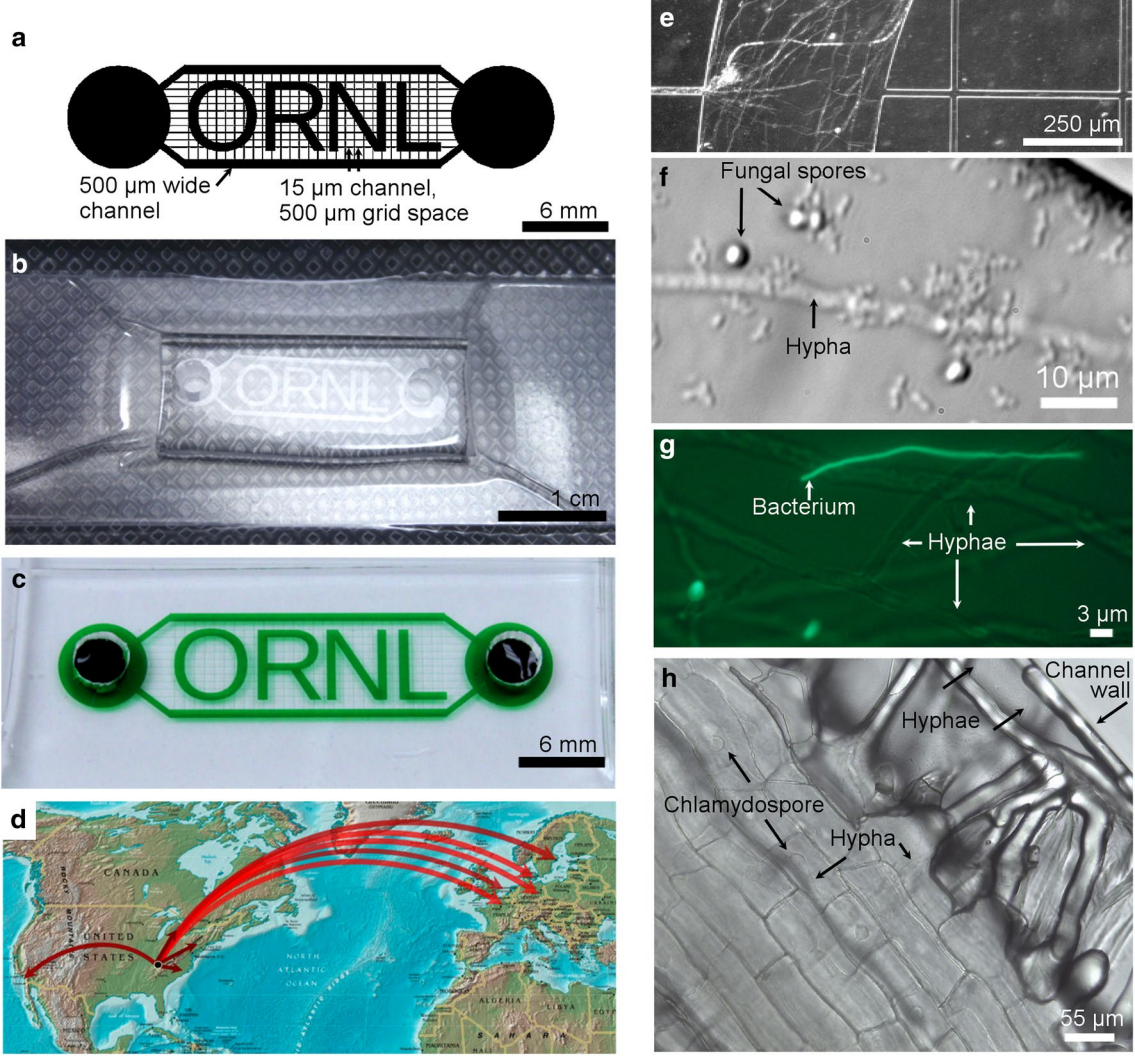
Ready-to-use microfluidics for studying the biology of branched structures. **a** Schematic design of a four-chamber (O R N L) microfluidic architecture with two end-wells for cultures (6 mm circles). The interconnecting channel grid dimensions are shown, 15 µm wide channels spaced by 500 µm gap. Boundary channels framing the four chambers and grid are 500 µm wide. **b** A sterile vacuum-packaged microfluidic chip ready for shipment, and ready to use. **c** An ORNL microfluidic chip with green dye in channels. **d** Map showing distribution of 28 ready-to-use ORNL chambers to 18 potential collaborators for fungal and other branched biology research (United States: Michigan, New York, North Carolina, and California. Europe: France, Germany, Netherlands, Denmark, Hungary, Sweden, and Switzerland0. **e**–**h** Results from implementing ready-to-use microfluidics in collaborative labs. **e** Rapidly growing *M. elongata* AG77 navigates the peripheral channels to fill the ORNL chambers of the device. **f** Microfluidic co-culture of *Neurospora crassa* and *Pseudomonas fluorescens* in microfluidic cultures (bacteria 1 μm, hypha 3 μm). **g** Merged image of bright field and fluorescence image of *Paraburkholderia caribensis* and *M. elongata*. **h** *Nicotiana attenuata* root grown in the presence of endophytic fungi, *M. elongata* NVP64+ ; fungal hypha and chlamydospores are observed within the plant root

To improve access to microfluidics for fungal-bacterial interaction studies by microbiologists, we developed a universal chamber-grid architecture amenable for culturing fungi, bacteria, or bacterial-fungal co-cultures. We vacuum-packed sterile PDMS-based microfluidics for distribution to potential collaborators through a workshop on bacterial-fungal interactions. The ORNL microfluidic system is designed to contain two outer boundary channels and four central chambers in the formation of letters, “ORNL” (Fig. 5a). Upon removal of the microfluidic chamber from the vacuum-sealed pouch, the channel array is primed by adding fluid to all ports of the microfluidic channel system (Fig. 5b, c). 28 sample chambers were distributed to 18 fungal biologists within the United States (Michigan, New York, North Carolina) and throughout Europe (France, Germany, Netherlands, Denmark, Hungary, Sweden, and Switzerland) (Fig. 5d). Of the responses, at least 13 samples from 9 collaborators yielded 6 successful cultures. Figure 5e–h demonstrates a sample of the range of results achieved from successful cultures in microfluidics primed after removal from the vacuum package. Of the 7 unsuccessful reports, three samples only partially filled, and three containers reportedly lost vacuum by handling, and one sample was neglected until long after the ‘best use by date.’ A discussion on the contributing factors competing with complete successful deployment is contained in the following discussion section.

Successful implementation of ORNL-chamber microfluidics to visualize fungal-bacterial and fungal-plant interactions was achieved through collaborative exchanges (Fig. 5f–h). Bacterial-fungal interactions can occur between *Neurospora crassa* and *P. fluorescens* In5 (Fig. 5f). The bacterium was isolated from Greenlandic soil which is naturally suppressive to plant infections by fungal pathogens [51]. Since some antagonistic bacteria are known to attach to fungal hyphae and parasitize the fungus without penetrating the hyphae [53] it was of interest to investigate if the In5 isolate attach to fungi*. Neurospora crassa* was selected for growth in the chamber for its fast growth and wide hyphae. As can be seen in Fig. 5f the bacteria may attach to, or highly associate with, the fungus. Further studies are warranted to determine if *N. crassa* forms microcolonies along the growing hypha.

With chambers and boundary channels available for high-resolution imaging, the ORNL microfluidic devices were used to visualize fine-scale interactions between a plant growth promoting bacterium *Paraburkholderia caribensis* and *Mortierella elongata* NVP64+, a fungus that naturally hosts endobacteria belonging to Burkholderiaceae. We were specifically interested in whether *Paraburkholderia caribensis* is able to enter the mycelium of *M. elongata*. A co-culture of *M. elongata* and a GFP-transformed strain of *P. caribensis* were initiated in an ORNL chamber, each inoculated in their own port. For this microbial pair we did not see a strong interaction between partners, under these conditions at 10 days in culture; however, a number of fluorescent cells characterized by single long sinuous shape were observed (Fig. 5g) when co-cultured with *M. elongata*, in addition to the typical bacilli and diplobacilli morphologies prevalent with this bacterium. This was repeated and confirmed by fluorescence microscopy. This morphology of *P. caribensis* has not been observed previously, and it is not known what triggers this morphological switch in the bacterium.

To begin probing mechanisms of endophytic growth in *M. elongata*, we introduced germinated *Nicotiana attenuata* seeds into one end of the microfluidic wells of ORNL vacuum-packed microfluidics. The other of the two ports was inoculated with *M. elongata* NVP64. Within 1 week of growth we observed fungal hypha and chlamydospores, or vesicle-like structures, of *M. elongata* within the plant roots (Fig. 5h). Fungal growth within the root hairs was not observed in this sample suggesting that *M. elongata* may enter roots through epidermal cells.

To better visualize and quantify the rate of cytoplasmic streaming in M. *elongata* NVP64+, we used ORNL microfluidic devices. Reverse cytoplasmic streaming was observed in *M. elongata* NVP64+ using these microfluidic chambers, with cellular vesicles and contents moving in opposite directions (Additional file 4: Figure S3). Consistent with prior reports for cytoplasmic streaming in fungi [67, 68], clear cytoplasmic paths containing relatively fast velocity streaming and slow velocity streaming were observed, as evidenced by the black streaks running in straight diagonal and curved lines (Additional file 4: Figure S3).

## Discussion

In this work we sought to overcome the common frustrations present with adopting microfluidic technology that limits the successful incorporation of microfluidics into traditional biology. The highlight of this work extends beyond a mere description of our approach to improving the accessibility to microfluidics, but also includes results from successful tests of microfluidics from collaborative works. First, we briefly discuss our approach to overcome these barriers including helpful tips for new users of microfluidics. In the remaining discussion we delineate the benefits and implications of ready-to-use microfluidics. We also highlight the examples and illuminate future directions of research possibilities that can be realized by microfluidics made easier.

### Barriers to microfluidics in biology

Barriers to implementing microfluidics in science and research settings span a range of categories, from operational barriers (protocols and the degree of subject-matter or device sophistication) to technical barriers (facilities and equipment) to individual barriers (experience and access to collaborators). We introduce a process that aids in removing technical and operational barriers through our design and packaging process. Nevertheless, individual experience and repetition is still required to maximize the use of microfluidic systems in biological science.

Fluidic priming is a common operational barrier to implementing microfluidics. The process of loading the microfluidic channel with fluid without introducing bubbles is a common hurdle in the implementation of microfluidics in biological labs. Filling the channels with fluid while eliminating bubbles may seem to be a trivial problem to one trained in the field, but it can be a tremendous barrier to entry for the microfluidic novice. Other technical barriers such as available equipment and facilities (for example, house vacuum) can put new collaborators, beginning to work with microfluidics, at a significant disadvantage or discourage collaboration. Syringe pumps are not typically available within biological labs; therefore, other means must be employed to prime the channels with fluid, or significant time and finances are a needed to invest in new pumping systems. A reliable alternative to priming microfluidics without syringe or pneumatic pumps is to equilibrate the PDMS channels in a vacuum chamber, or desiccator, then upon releasing the vacuum, immediately supply fluid to the channels (*e.g.* water, cell culture media, food color dye, oil, solvents) [54, 69]. As the PDMS equilibrates with the local atmosphere, the material efficiently absorbs the air from the channels and is displaced by fluid from the fluid-filled ports. However, even for this simple approach, much time is spent solving this limitation on a lab-to-lab basis—regardless of distance between labs—due to the lack of vacuum supplies and equipment, and the variability in vacuum quality. House vacuum supplies, if available for degassing PDMS, are typically low vacuum (~ 70 kPa) and require long vacuum equilibration times to achieve enough drawing capacity to completely fill the channels with fluid. Vacuum pumps (> 80 kPa) may be available or can be purchased; however expensive equipment investments are typically unwarranted until the feasibility and promise of the microfluidic technologies have been established for collaborative investigators.

The design and architecture of a microfluidic system can potentially be a barrier to this enabling technology. While a detailed discussion on effective architectural elements for easily implementing microfluidic platforms is beyond the scope of this work, it should be noted that depending on the volume of the microfluidic architecture, house vacuum may be vastly insufficient in providing enough drawing capacity to completely prime microfluidic channels with fluid. As an alternative, inert gasses can be connected through specialized connections to push the fluid into the channels to displace bubbles through the material, here again, specialized supplies are needed.

To overcome common *technical barriers* that hinder new microfluidics experimentation, we sought to simplify the fluidic priming process by using a common vacuum-based approach that fills-on-demand. This inexpensive and commercially available packaging process allows for the retention of a vacuum potential within PDMS-based microfluidics for up to 2 weeks when stored under vacuum, thus preserving the ability to prime the microfluidics when returning the PDMS material to atmospheric conditions (Fig. 2). We also eliminated the need for the end-user to determine and incorporate sanitization processes for an unfamiliar technology; we pre-cleaned the microfluidics and included sanitization into the assembly, vacuum, and packaging process. We increased the *accessibility* to microfluidics by demonstrating a process that decreases the cost-of-investment and minimizes the steep learning curve. We disseminated microfluidics to potential collaborators for testing and use. Through our simple, inexpensive, effective, and easy-to-use process, academic labs can deliver a valuable microfluidic product for biologists to use for resolving the spatial and temporal dynamics of biological interactions (e.g. bacterial-fungal, bacterial-plant, and fungal-plant interactions).

### Benefits and implications

Microfluidics are ideally suited to benefit studies of branched biological systems and mixed community interactions, for they enable physically and chemically controlled microenvironments. In nature, branched structures are capable of extending into multiple spatial domains for resource exploitation (plants and fungi), signal transduction and integration (neurons and fungi), mechanical support (plants and fungi), mass transport (plants, fungi, neurons), and cellular/organismal adaptation and regulation [55, 56].

The robust and inert construction of microfluidics enables pre-priming, filling, and shipping of hydrated microfluidics, with or without cultures. Preconditioning of microfluidics may be useful for initiating cultures prior to shipment, or in collecting and preserving field samples.

Incorporating agar plugs (Fig. 3b) into microfluidics substantially delays drying and provides an anchor for fungal cultures. Additionally, agar can be poured around the periphery of the microfluidics within the dish to aid in hydrating the PDMS and moistening the chamber for long-term growth and observation without fluid spillage and shearing cells.

With our approach, potential users need only open and fill the microfluidic platform to prepare samples for high-resolution imaging and can forego learning the device fabrication, assembly, sanitizing, and other preparation processes. For example, we tested our vacuum preconditioning process before deploying to collaborators, we packaged and germinated *Arabidopsis* seeds on chip. The channels filled with fluid, the majority of seeds germinated down the channel, and the cultures remained sterile, all without an overall impact to the root length. From these tests, we learned that the ability to vacuum seal and viably store seeds in a microfluidic platform has the potential to greatly enhance collaborations within and across disciplines. The low cost and portability of the Food Saver system also allows for field researchers to sow collected seeds or spores directly into a microfluidic platform and vacuum package for later use, thus preserving the seed with native microbes.

The process of preparing inoculated cultures in microfluidics saves time and eliminates initial barriers to implementing microfluidics. For example, *Laccaria bicolor* is a slow growing fungus and the culture can begin to be established before or during shipment. To confront this challenge, we prepared microfluidics on glass-bottomed dishes, then primed with culture media and agar, inoculated the central compartment with *Laccaria* fungi prior to sealing the dishes and shipping the samples to collaborators for bacterial inoculation and observation of bacterial-fungal interactions. After sustained development of *Laccaria* in the microfluidics during shipment and extended culture, transgenic bacteria were introduced and documented with microscopy (Fig. 3g). Biofilms formed on both bacterial-fungal co-cultures within 1 month and improve access to imaging bacterial-fungal interactions. While beneficial for some samples, this process is not suitable for all experimental conditions, cells, or organisms. Indeed, most biological samples cannot be maintained so easily during transport, (mammalian cells for example); therefore, a more ready-to-use approach is needed to expedite collaborative exchanges by overcoming initial barriers to implementing microfluidics in biology.

As an alternative to preparing cultures for transport, we propose a ready-to-use approach where microfluidics can be preconditioned dry, with vacuum, or hydrated and conditioned with agar to prevent drying and to provide an immediate-use product for the recipient to inoculate.

### Deployment of vacuum-packed microfluidics

After characterizing the ‘ready-to-use microfluidics,’ we prepared spoke-wheel microfluidics and characterized the filling time as a function of post-packaging storage time. From these samples, we determined a 2-week window for a ‘best-use by’ date. To deploy ready-to-use-microfluidics with this preparation process, we fabricated an “ORNL” microfluidic architecture for testing and use by potential domestic and international collaborators. From our experience, the best process for loading chambers and successful culture is to initiate microfluidic priming within a 2-day shipping window. Longer shipments or travel may increase the time to fill, or rate of failure for this microfluidic preparation. One possible explanation for increased fill times or incomplete filling after 2 weeks in vacuum storage includes handling-induced loss of vacuum to the packaged system. Another possible explanation includes, age- and environmental-related (temperature) aging or stiffening of the elastomeric material. For example, twenty-eight sample chambers were deployed in response to interests communicated at a bacterial-fungal interactions workshop. Factors influencing the rate of successfully obtained cultures include, travel time, sample handling and storage during transit, and the learning curve for handling and filling microfluidics. Notwithstanding these possible limitations, some users reported that the devices were simple and easy to use, while others needed more replicates to successfully implement microfluidics in their experimental system. In this regard, we cannot overlook the procedural requirements of the biological system being integrated into the microfluidic chip. Culturing cells at room temperature without specialized gaseous environmental culture conditions (e.g. fungal cultures) may be easier and more forgiving, thus providing a higher apparent ‘success rate’ than trying to implement microfluidics with a biological protocol were time, temperature, and gas concentrations are all critical (e.g. anaerobic cultures and mammalian cell cultures). By extension, learning to use microfluidics in a biological safety cabinet is more challenging than working with them on a benchtop. Overall, we were pleased with the ability of this packaging processes to accelerate collaborative research and to quickly achieve productive results. It is our goal that this vacuum-packing process can be adopted and decrease the barriers to implementing microfluidics by providing a process to get microfluidics in-hand to end-users for testing and implementing in biological science.

### Focus areas and future directions

Future research on fungal-bacterial interactions with bacteria could include fluorescent vitality stains for assessing fungi and bacteria, the use of fluorescence reporters for monitoring gene expression activity in microbes, or the use of chamber doors for sampling cells, nucleotides or metabolites from interacting tissues. Of special interest would be to investigate (1) if the architecture of the chamber can influence the outcome of a biological interaction related to previous studies, (2) how bacteria and fungi enter and grow within plant roots, (3) how environmental conditions influence cytoplasmic flow of cellular contents, or the colonization and community structure of the rhizosphere.

Microfluidic platforms have proven to be indispensable tools for probing processes of cellular function, organismal behavior, and environmental interactions. While biomedical disciplines have greatly benefited from the discoveries enabled by microfluidics [22], botany and mycology are ripe for microfluidic-enabled discoveries and solutions. Focus areas include, but are not limited to, hyphal chemotropism, fungal pathogenecity, tripartite interactions of the rhizosphere. More specifically, bacterial-fungal interaction studies are also in their infancy, as evidenced by much higher resolution studies [70–72]. While microfluidics offer the opportunity to engineer microcosms for probing the physical, chemical and biologicals aspects of multispecies interactions, these devices are convenient (but not required) for such imaging studies in general. Plant-fungal mutualism studies offer ample promise, and plant-bacteria interactions are valuable as well in medicine as for bioenergy, food crop production, and purifying natural resources [57–60].

Prior to deploying vacuum-equilibrated microfluidics for studying the biology of branching structures, we developed an architecture that enables low-density, high resolution access to branched hyphae for fungal-bacterial interaction studies. Spoke-wheel microfluidics for ectomycorrhizal bacterial-fungal interactions were tested in-house before shipping to international colleagues for implementation (Fig. 3). The purpose of our microfluidic design was to restrict the hyphal growth region to a large fluidic area while maintaining a low microchannel ceiling (11 µm) that confines the biological observations to a stationary fluidic microenvironment for imaging. This work demonstrates the ability to accelerate studies of defined microcosms within microfluidic platforms by incorporating fabrication and sterilization processes into the packing process for shipping and sharing.

## Conclusions

We have characterized and deployed an affordable, readily available, and inexpensive process that solves the priming challenge, for new collaborators, by storing sterile dry vacuum-equilibrated microfluidics in a vacuum-sealed pouch for easy fluid priming, anytime, anywhere. This preparation and packaging process allow the end user to achieve fluid-primed microfluidics without the need for pumping systems. The need for fluidic priming at the fabrication source is eliminated, the shipment of fluids is avoided, and the ease of priming microfluidics is achieved. This process requires the fabrication source to have access to equipment for sterilizing (UV, plasma, autoclave), generating relatively high vacuum (< 81 kPa), and maintaining sterility during the packaging process (biosafety cabinet). The end user specifies and supplies the fluids, thereby alleviating the supplier from the need to fluid-match recipes, requirements (pH), or conditions (prevent fluid aging or contamination).

We present a process that accelerates the adoption of microfluidics in labs lacking experience with the technique. The adaptation of challenging microtechnological methods across discipline boundaries can further accelerate the development and implementation of defined microcosms and engineered niches to better resolve fungal, bacterial, and plant interactions. By extension, microfluidics could also allow for the extraction or injection of biological contents for biochemical analyses and genome regulation studies, respectively. Breaking down scientific barriers through technological simplifications enables studies that have the power to move science forward toward resolving mechanisms of complex and pressing biological problems (e.g. microbial symbiosis or pathogen-host interactions) [14].

## Additional files

**Additional file 1.** Supporting methods.

**Additional file 2.** Maturation of bacterial-fungal interactions in microfluidics.

**Additional file 3.** Dynamics of bacteria along the ‘hyphal-highway’ and cytoplasmic streaming within the mycelium.

**Additional file 4.** Bidirectional cytoplasmic streaming in fungal hyphae within microfluidic culture.

## Data Availability

The datasets used and/or analyzed during the current study are available from the corresponding author on reasonable request.
