# Supplementary material for: Increasing access to microfluidics for studying fungi and other branched biological structures
Source: Fungal Biol Biotechnol. 2019 Jun 10;6:1. doi: 10.1186/s40694-019-0071-z (PMC6556955; doi:10.1186/s40694-019-0071-z)
Supplement: Supplementary file 1 — Additional file 1. Supporting methods. [file 40694_2019_71_MOESM1_ESM.docx]

**Additional File 1: Supporting Methods**

**Microfluidic fabrication**

Soda-lime masks were printed in-house for conventional photolithographic processes.^1^ Briefly, clean four-inch silicon wafers were spin-coated with MicroPrime P20 adhesion promoter (Shin-Etsu Microsci). SU-8 photoresist (MicroChem, Westborough, MA) was spun onto the wafer to the respective thickness, then soft baked, exposed, and post-exposure baked according to manufacturer’s recommendations. The patterned wafer was developed with SU-8 developer, rinsed with isopropyl alcohol, distilled water, and dried with a stream of nitrogen gas. Immediately prior to silane treatment, the patterned wafer was cleaned with air plasma (1-2 min). Silane exposure (trichloro(1H,1H,2H,2H-perfluoro-n-octyl)silane, 85 °C, 60-120 min) coated the wafer with a non-stick coating to prevent PDMS adhesion to the patterned wafer during replicate molding. Liquid PDMS (prepolymer-to-curing agent, 10:1) was poured onto the wafer, degassed, baked (70 °C) and removed from the wafer. Microfluidic replicates were trimmed, and wells were cut with biopsy punches.

For PDMS microfluidic channels on microscope slides, the trimmed microfluidic replicates were cleaned with 3M Magic tape immediately prior to assembly. To assemble microfluidics on slides, tape-cleaned PDMS replicates and glass slides were air plasma treated (1-2 min) then immediately brought into conformal contact to seal the channels, then oven-baked (70°C, 20 min). Channels on glass were autoclave sterilized in a sealed metal container then transferred to a vacuum chamber in a sterile biosafety cabinet.

For microfluidics in glass-bottomed Petri dishes, the trimmed and tape-cleaned microfluidic replicates were air plasma treated, then sealed with conformal contact, then baked (65 °C, 20 min). Assembled channels in dishes were sterilized with UV irradiation (3 min each side) using a UV crosslinker oven (UV Stratalinker 2400, Stratagene). The sterilized dishes with channels were closed in the UV sterilizer and transported to a biosafety cabinet.

**Detailed Cell Culture Methods**

*Laccaria bicolor.* Vacuum equilibrated spoke-wheel microfluidics on coverslips were pre-loaded with P20th- media in a Petri dish and set aside (20 min) until all bubbles were removed from the channels by the vacuum-treated PDMS. Media-filled microfluidics were briefly placed on a hotplate (50°C) to fill with liquid agar by pipetting hot agar into the central well of the wheel and aspirating out of the accessory port. The microfluidics were removed from the plate and allowed to cool and gel. The microfluidics were then UV-sterilized for 2 min. Agar was poured around the periphery of the microfluidics to minimize evaporation during culture, transportation and imaging. To inoculate the microfluidics, a plug was removed from the center of the chip and replaced with an agar plug containing *L. bicolor* S238N. Inoculated fungi were maintained in culture during transportation, and after bacterial (*Pseudomonas fluorescens* BBc6) inoculation for at least 1 month.

*Neurospora crassa.* Microfluidic co-culture of *N. crassa* and *P. fluorescence* In5 ^2^ was achieved by removing the microfluidic device from the vacuum-sealed pouch. The entire device was placed in a Petri dish, then diluted PDB medium containing fungal antagonistic bacteria was pipetted into one of the wells and media with *N. crassa* spores were loaded into the other end of the microfluidic chip. Cultures were maintained and observed by Professor Stefan Olsson. Images were acquired using digitally enhanced asymmetric illumination using very low light levels for cell viability.^3^

*Arabidopsis thaliana Columbia* line (col-0) seeds were surface sterilized for 10 minutes using 30% bleach and 0.1% Triton X solution and washed 4 times in sterile water to remove excess detergent. The sterile seeds were transferred to a refrigerator (4°C, < 1 week) to synchronize germination. After stratification, seeds were individually transferred into the inlet of a sterilized plant-on-a-chip device using a pipette and placed under vacuum for approximately 30 minutes. Plant-chips were removed from the vacuum chamber and each device was immediately vacuum-sealed using a Food Saver vacuum packaging unit. The sealed devices were then covered in aluminum foil to prevent light-induced germination, and stored on a bench top for the duration of either 7 or 14 days. When the storage period ended, the packaging was cut open and the plant-chip was submerged in a Petri dish filled with liquid 1/4x Murashige-Skoog media. Media was pulled through the device using a pipette in the outlet channel and then the device was transferred to a new Petri dish and placed vertically in a 12-hour light/dark cycling growth chamber to promote germination and root growth down the channel. To maintain an aseptic environment, the root’s growth was measured daily by imaging the plant-in-chip with an Olympus inverted microscope through the bottom of the petri dish.

*Mortierella elongata* strain AG77 (Zygomycota, Mortierellomycotina) was grown on 1.5% malt extract agar (MEA) as described previously.^4^ Plates were stored at ambient temperature on parafilm-sealed MEA plates to avoid contaminants. Pre-vacuumed microfluidic devices were loaded with p20 media lacking thiamine, as described previously.^5^ *M. elongata* was grown on MEA plates for 2 days before a 1-mm diameter agar plug was inserted into the inoculation ports. Microfluidic devices inoculated with *M. elongata* were incubated at ambient temperature for 2 days and visually examined for hyphal growth and movement through channels. Images were taken on an Axio observer inverted microscope using a Hamamatsu ORCA ER Series C4742-95-12 digital camera.

*Nicotiana attenuata* and *M. elongata* NVP64+ were co-cultured in a microfluidics device. Vacuum-sealed microfluidic devices were removed from their pouch and were loaded with Murashige Skoog broth containing 1% dextrose. *N. attenuata* seeds were sterilized and germinated as previously described.^6^ A single germinated *N. attenuata* seed was placed in one of the wells. *M. elongata* NVP64+ was grown on MEA for 2 days and a 0.5 cm^2^ plug of MEA colonized by actively growing *M. elongata* was used to inoculate the microfluidics device by placing top-side down over the well on the opposite side of the device. Plates were observed daily over a 10-day duration on a Leica DM750 microscope with an ICC50 HD digital camera.

*Paraburkholderia caribensis* and *M. elongata* microfluidic co-culture was achieved by first removing microfluidic channels from their vacuum-sealed pouch and immediately filling wells with liquid malt extract broth, and finally inoculating the microfluidics for culture and analysis. MiniTn7-GFP tagging was achieved according to previously published protocols.^7^ GFP-tagged *P.* *caribensis* XV bacteria were first cultured in malt extract broth at a concentration of 1x10^6^ CFU/mL (.001 OD-600) and a 20 μl cell suspension was used to inoculate 1 side of the microfluidic platform. A 0.5 cm^2^ plug of 2 day old actively growing *M. elongata* NVP64+ was used to inoculate the microfluidics device by placing top-side down over 1 the well on the opposite side of the ORNL device. Cultures were observed at one, two and three days post inoculation on an Olympus IX71 microscopy with an Olympus DP71 digital camera. The chamber was kept hydrated by placing it in a Petri dish containing autoclave-sterilized ddH_2_O.

*Neuronal culture and staining.* Neurons from male and female P0-2 mouse frontal cortex were dissociated with papain and re-suspended in Neurobasal/GS21/Glutamax medium at a density of 3 x 10^6^ neurons per ml with 10 μl of cell suspension added to the central port of the microfluidic device.^8^ Cells were allowed to adhere for 10 mins before adding 40 μl of additional media. Cells were fixed at DIV 4 in 4% paraformaldehyde, 4% sucrose in PBS at 4°C for 10 min. Neurons were permeabilized with 0.25% Triton X-100 for 5 min, blocked with 10% BSA, and then incubated with PSD-95 (K28/43, 1:1000; NeuroMab) and VAMP2 (104-202, 1:1000, Synaptic Systems) primary antibodies and Alexafluor-488 and Cyanine-3 secondary antibodies diluted in 3% BSA. Imaging of neurons was carried out using a Nikon C2Si (NIS-Elements software v4.5) laser scanning confocal microscope with a 60X Plan Apo oil immersion objective (1.4 NA).

**Literature Cited**

^1^ Millet LJ, Lucheon J, Standaert RF, Retterer ST, Doktycz MJ. Lab Chip. 15(8):1799–811 (2015).

^2^ C.F. Michelsen, J. Watrous, M.A. Glaring, R. Kersten, N. Koyama, P.C. Dorrestein, and P. Stougaard, MBio **6**, e00079 (2015).

^3^ B. Kachar, Science. **227**(4688), 766-768 (1985).

^4^ J. Uehling, A. Gryganskyi, K. Hameed, T. Tschaplinski, P.K. Misztal, S. Wu, A. Desirò, N. Vande Pol, Z. Du, A. Zienkiewicz, K. Zienkiewicz, E. Morin, E. Tisserant, R. Splivallo, M. Hainaut, B. Henrissat, R. Ohm, A. Kuo, J. Yan, A. Lipzen, M. Nolan, K. LaButti, K. Barry, A.H. Goldstein, J. Labbé, C. Schadt, G. Tuskan, I. Grigoriev, F. Martin, R. Vilgalys, and G. Bonito, Environ. Microbiol. **19**(8):2964-2983 (2017). doi: 10.1111/1462-2920.13669.

^5^ A. Müller, K. Volmer, M. Mishra-Knyrim, and A. Polle, Front. Plant Sci. **4**, 332 (2013). doi: 10.3389/fpls.2013.00332.

^6^ T. Krügel, M. Lim, K. Gase, R. Halitschke, IT. Baldwin, Chemoecology **12**(4), 177-183, (2002). <http://hdl.handle.net/11858/00-001M-0000-0012-ADE2-3>

^7^ T.K. Teal, D.P. Lies, B.J. Wold, and D.K. Newman, Appl. Environ. Microbiol. **72**(11):7324-30 (2006).

^8^ Y. Chen, B. Stevens, J. Chang, J. Milbrandt, B.A. Barres, and J.W. Hell, J Neurosci Methods **171**(2):239-47. doi: 10.1016/j.jneumeth.2008.03.013. (2008).
