# Supplementary material for: Increasing access to microfluidics for studying fungi and other branched biological structures
Source: Fungal Biol Biotechnol. 2019 Jun 10;6:1. doi: 10.1186/s40694-019-0071-z (PMC6556955; doi:10.1186/s40694-019-0071-z)
Supplement: Supplementary file 2 — Additional file 2. Maturation of bacterial-fungal interactions in microfluidics. [file 40694_2019_71_MOESM2_ESM.docx]

**Additional File 2: Figure S1**


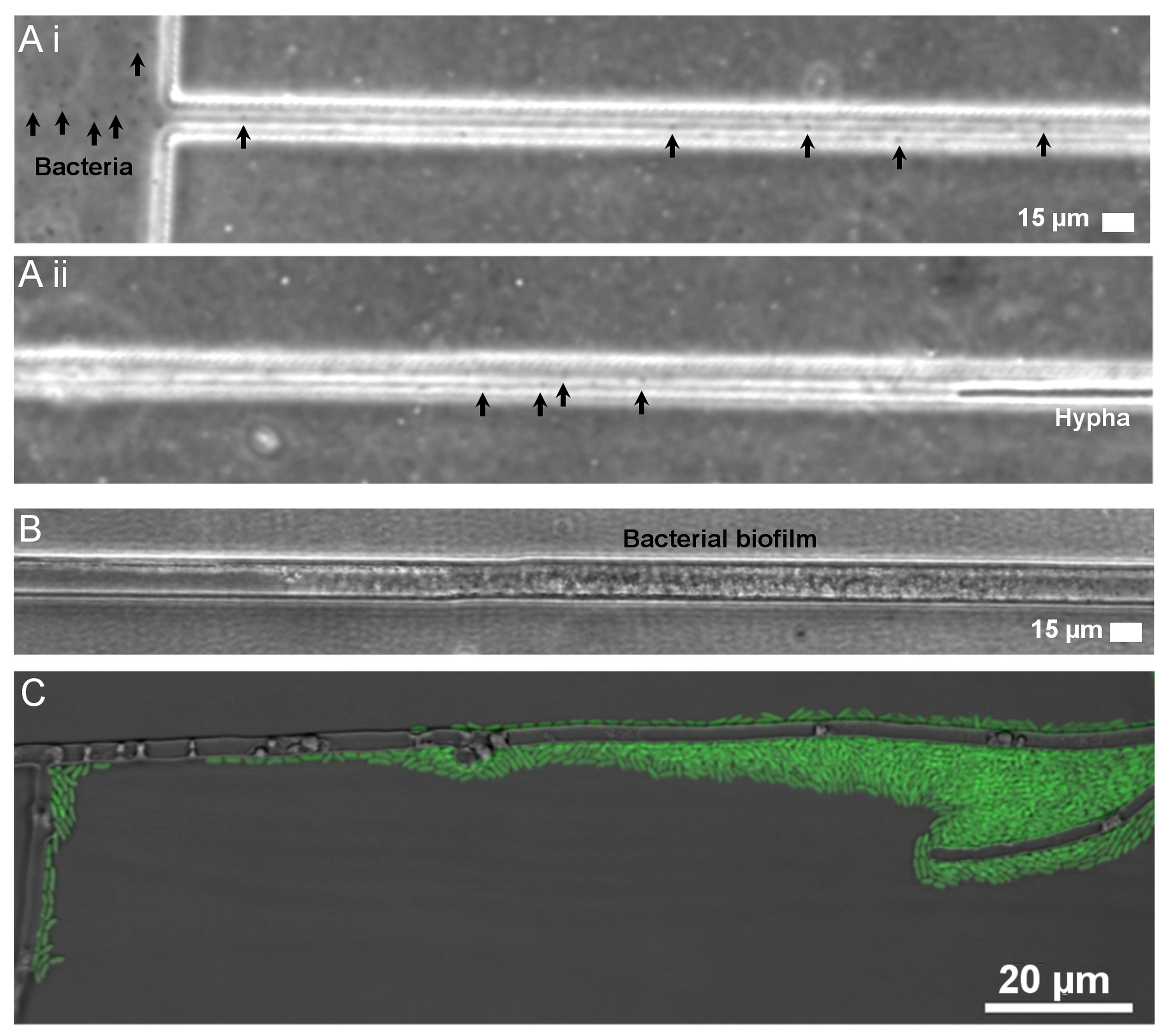


**Figure S1.** Maturation of bacterial-fungal interactions in microfluidics. (A(i-ii)) Pair of images showing individual *Pseudomonas fluorescens* GM41 bacteria and *Laccaria bicolor* S238N hypha within a single secondary microfluidic channel of the spoke-wheel design. The top image shows bacteria in the outer peripheral channel and secondary channel, the bottom image is a continuation of the same channel and shows the hypha and bacteria. (B) Biofilms readily form within most secondary channels, with or without fungi. (C) In the same device architecture, *Pseudomonas fluorescens* BBc6 biofilm-like accumulation on ectomycorrhizal fungi (*L. bicolor* S238N) 16 hours after bacterial inoculation.
