# Supplementary material for: Increasing access to microfluidics for studying fungi and other branched biological structures
Source: Fungal Biol Biotechnol. 2019 Jun 10;6:1. doi: 10.1186/s40694-019-0071-z (PMC6556955; doi:10.1186/s40694-019-0071-z)
Supplement: Supplementary file 3 — Additional file 3. Dynamics of bacteria along the ‘hyphal-highway’ and cytoplasmic streaming within the mycelium. [file 40694_2019_71_MOESM3_ESM.docx]

**Additional File 3: Figure S2**


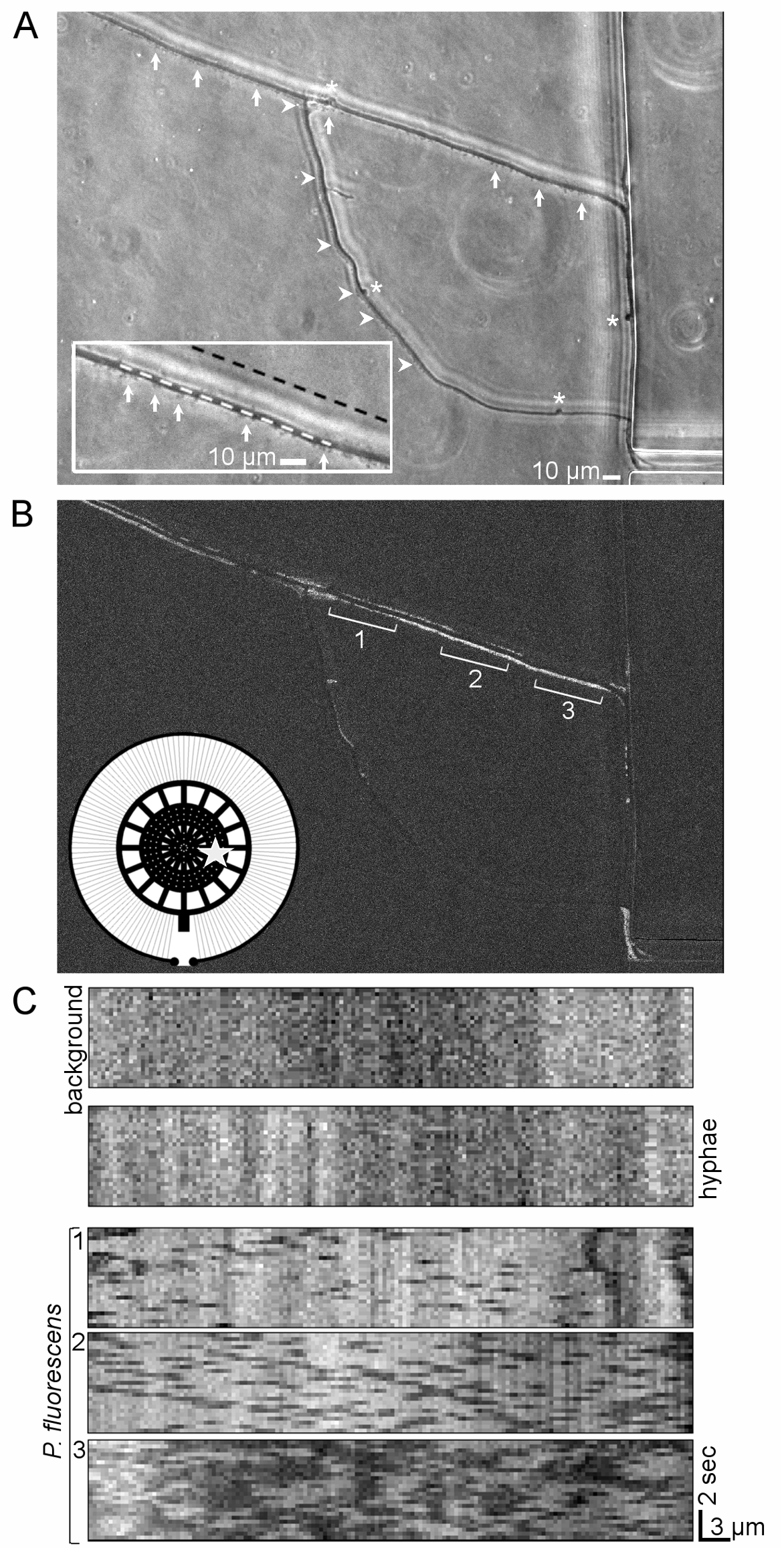


**Figure S2.** Dynamics of bacteria along the ‘hyphal-highway’ and cytoplasmic streaming within the mycelium. (A) *Pseudomonas fluorescens* GM41 colonize and migrate along the surface of *Laccaria bicolor* S238N fungal hyphae isolated using the ‘spoke-wheel’ microfluidics. Inset: dashed-line traces for hyphal and background kymographs. Arrows: regions of high bacterial motility; chevrons: colonized regions with low bacterial motility; Asterisks: fungal cell-cell clamp connections. (B) Standard deviation image of the time-lapse stack (25 images, 6 sec) shows a 2-dimensional view of regions with high bacterial motility which appear here as white edges lining the hyphae. The marked segments (1, 2, 3) of the hyphae designate areas of low, moderate, and high bacterial motility, respectively. (C) Kymographs representing time-lapse data of bacterial motility (black rectangles of the pixel grid) near the surface of the hyphae for areas marked in (B). The background line trace is from the fluid-filled channel near the hyphae; hyphal line trace is from the center of the hypha, and three highway traces of *P. fluorescens* GM41 depict abundance of bacteria swimming near the hyphal surface for low (1), moderate (2), and high (3) activity.
