# Supplementary material for: Increasing access to microfluidics for studying fungi and other branched biological structures
Source: Fungal Biol Biotechnol. 2019 Jun 10;6:1. doi: 10.1186/s40694-019-0071-z (PMC6556955; doi:10.1186/s40694-019-0071-z)
Supplement: Supplementary file 4 — Additional file 4. Bidirectional cytoplasmic streaming in fungal hyphae within microfluidic culture. [file 40694_2019_71_MOESM4_ESM.docx]

**Additional File 4: Figure S3**


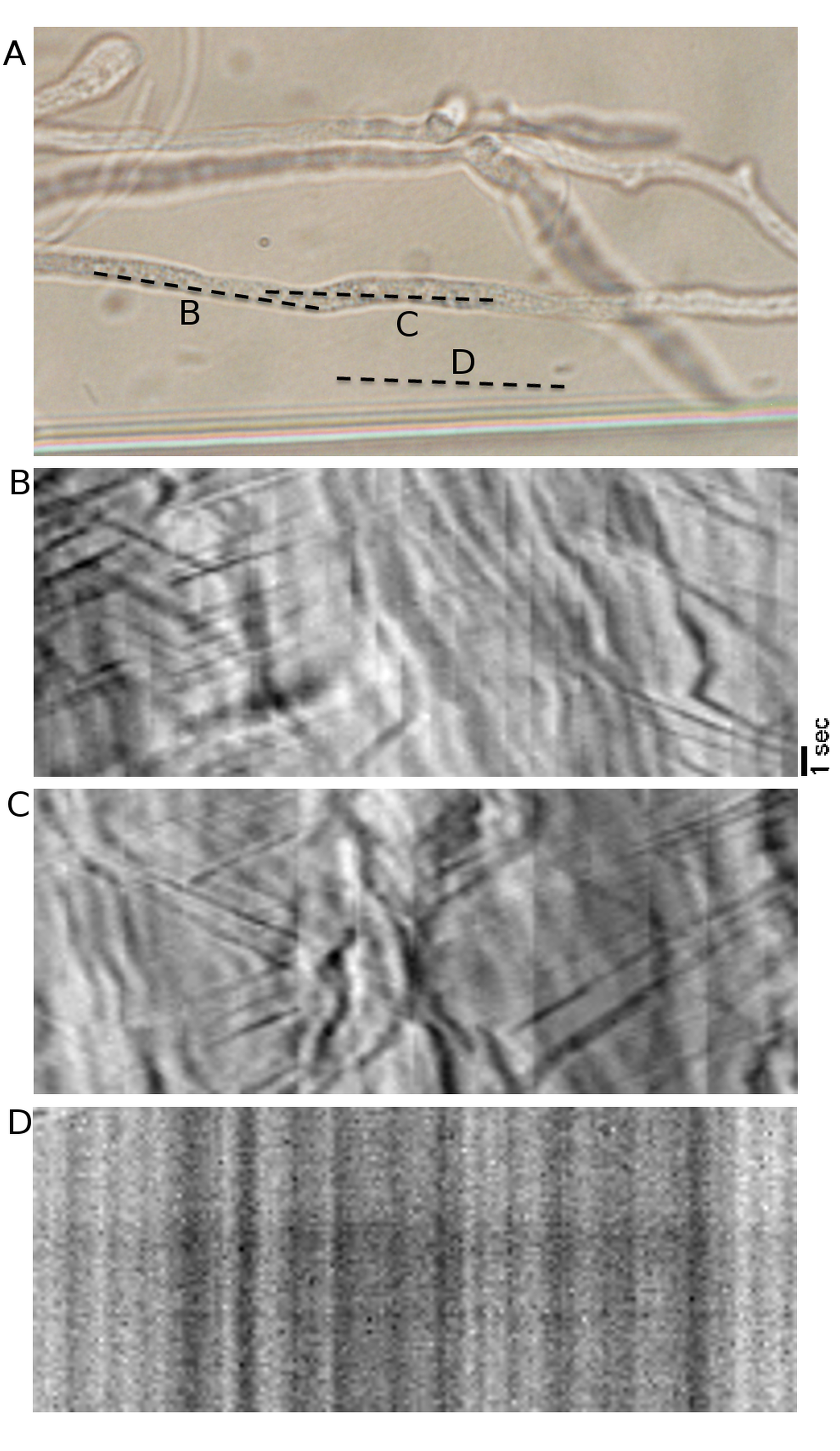


**Figure S3.** Bidirectional cytoplasmic streaming in fungal hyphae within microfluidic culture. (A) *M. elongata* grows as coenocytic mycelium and is characterized by cytoplasmic streaming. Line traces through the time-lapse image stack are used for generating kymographs. (B-C) Kymographs display the bidirectional cytoplasmic streaming observed in culture identified by black diagonal and curved lines. (D) Kymograph of the fluid-filled channel without hyphae. The black dashed lines (panel A) mark the area of the cytoplasm for respective kymographs and channel background.
